# Sub-Second, Sensitive and Multispecies Detection of Volatile Organic Compounds Using a Mid-Infrared Broadband Supercontinuum Source and Upconversion Spectrometer

**DOI:** 10.1177/00037028261446656

**Published:** 2026-05-04

**Authors:** Yueyu Lin, Paola Formica, Roderik Krebbers, Amir Khodabakhsh, Simona M. Cristescu

**Affiliations:** 1 Trace Detection Laboratory, 226184 Institute for Molecules and Materials, Radboud University, Nijmegen, The Netherlands; 2 Department of Physics, 9295 University of Bari, Bari, Italy

**Keywords:** Volatile organic compounds, VOCs, mid-infrared, MIR supercontinuum source, upconversion spectroscopy, multispecies gas detection

## Abstract

Reliable monitoring and analysis of volatile organic compounds (VOCs) are crucial for environmental monitoring and human health. In this work, we report a system for fast, sensitive, and multispecies detection of VOCs by combining a fiber-based broadband mid-infrared (MIR) supercontinuum (SC) source with an upconversion spectrometer. The system’s performance in terms of species identification accuracy, robustness against inter-species interference and real-time multi-species detection capability was evaluated by monitoring and tracing the evaporation dynamics of three VOCs, i.e., acetone, ethanol, and α-pinene. We achieved detection limits of 30 parts per million per meter (ppm·m), 5 ppm·m, and 0.6 ppm·ּּm in 1 second for these three compounds, respectively. Furthermore, the evaporation behavior of individual compounds from a mixture was investigated to demonstrate the system’s capability for dynamic multicomponent analysis.

## Graphical abstract

**Graphical abstract:**
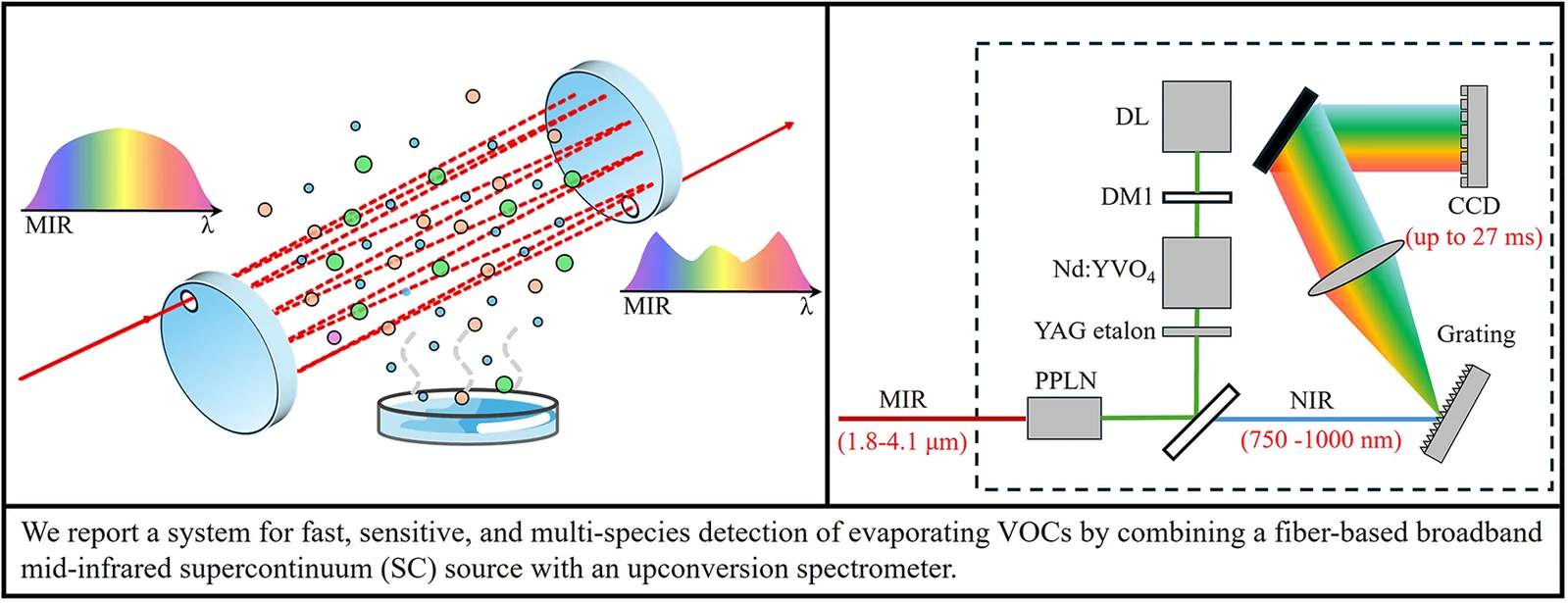
This is a visual representation of the abstract.

## Introduction

Volatile organic compounds (VOCs), a subset of organic compounds characterized by high vapor pressure and low water solubility, are widely released and distributed in all kinds of environments. There are two sources of VOCs: natural processes and various human activities in contemporary society.^1^ In natural processes, most VOCs are biogenic, largely emitted by plants. These VOCs are released as a result of metabolic activities, playing a crucial role in facilitating interactions with the environment.^2^ Apart from natural processes, human activities in both industrial production and daily life contribute significantly to anthropogenic sources, including the use and production of fossil fuels, solvents in coatings, paints, and inks, compressed aerosol products, biofuels, and biomass combustion.^3,4^ The chemical transformation of VOCs can produce highly reactive volatile substances, which can contribute to the increased pollution rate and even lead to climate change effects.^5,6^ Meanwhile, indoor VOC exposure has become an increasing concern compared with outdoor atmospheric pollution.^7–9^ As people spend most of their time in indoor environments such as offices, workstations, and residences, they are more likely to be exposed to numerous VOCs that originate from household materials or finishing products. Some VOCs are released directly indoors, and some are produced through chemical reactions.^10,11^ It was discovered that the total concentration of all VOCs indoors can be up to five times higher than that of the outdoor environment,^12^ which can lead to various health conditions, such as respiratory and cardiovascular diseases.^13^ Accurate, reliable, and timely information on VOCs, including their types, concentrations and/or spatial distribution, is crucial for understanding their impacts on both atmospheric pollution and human health.

Gas chromatography–mass spectrometry (GC-MS) is a commonly used method in detecting VOCs due to its high sensitivity and resolution, which allows for precise identification and quantitative analysis of VOC components.^14^ However, the bulky, costly equipment and required highly trained personnel complicate real-time online monitoring. In addition, sampling, transporting, and storing would increase the risk of sample degradation through contamination and adsorption. In recent years, optical sensing, especially in the infrared (IR) wavelength range, has developed rapidly in VOCs detection due to its better selectivity, lower instrumental drift, and faster response, with the additional benefit of being non-destructive. Most organic substances, including VOCs, exhibit specific and strong vibrational absorption characteristics in the IR region, especially at mid-infrared (MIR) range (2–20 μm). The fundamental vibrational transitions of most VOCs occur in the MIR, so-called “fingerprint region”.

Mid-infrared broadband absorption spectroscopy is a powerful and versatile technique for VOC detection. Based on Beer–Lambert Law, absorption spectroscopy enables the detection of molecular species with high sensitivity and selectivity. Compared to narrowband spectroscopy, broadband spectroscopy covers a wider range of the spectrum, capturing characteristic spectral features leading to reliable multispecies detection. Fiber-based MIR supercontinuum (SC) sources are replacing traditional thermal light sources as a new class of laser-based emitters for broadband MIR absorption spectroscopy, owing to their high brightness, broad spectral coverage, and spatially coherent output.^15–18^ A current challenge in broadband infrared spectroscopy is achieving fast and cost-effective spectral acquisition. Fourier transform (FT) spectroscopy and grating-based spectroscopy are widely used in broadband molecular spectroscopy, achieving a large spectral coverage and high spectral resolution. FT spectroscopy, based on a Michelson interferometer, relies on a path length difference induced by a mechanical movement, resulting in a limited acquisition speed in the order of seconds. For the grating-based spectroscopy, the grating disperses the light into a wavelength-dependent angle. Integrating the grating with a servomotor-driven galvo scanner enables precise spatial scanning and real-time spectral detection using a single-point photodetector, but the acquisition speed is still limited by the mechanical movement of the scanner.^19^ The acquisition speed can be significantly shortened by replacing the rotating grating and single-point detector with a fixed grating and a photodetector array. However, sensitive MIR photodetector arrays on the market are not readily available or mature. An alternative approach is upconversion spectroscopy, in which the MIR spectrum can be converted into near-infrared (NIR) through sum-frequency generation with a high-power pump laser in nonlinear crystals, enabling detection with mature NIR photodetector arrays.^20,21^ While this approach overcomes the challenge of fast MIR gas detection,^20,22^ another important issue arises from the way VOCs interact with the optical system itself, which is the tendency of VOCs to stick to the measurement cells. Considering this adsorption and possible contamination occur during the sampling and transporting of VOCs, an open gas cell can be employed to replace the traditional closed gas cell and form an open detection region. An open gas cell, consisting of two concave mirrors without any enclosure, not only enables multiple passes of the beam to increase detection sensitivity, but also allows the VOCs to move freely through the detection area, minimizing the contamination and adsorption associated with sampling lines and closed gas cells.

In this study, we report a configuration of a MIR SC source, an open multipass cell, and an upconverter spectrometer for VOCs detection. Three VOCs, i.e., acetone, ethanol, and α-pinene, were selected and measured during their evaporation. We evaluated the selectivity during the measurement for each of these compounds, and, simultaneously, retrieved the concentration of the ambient methane (CH_4_) and water vapor (H_2_O). Furthermore, we demonstrated the system’s ability for interference-free multispecies detection by tracing the evaporation of individual compounds from a mixture.

## Experimental

### Materials and Methods

**Figure 1.**
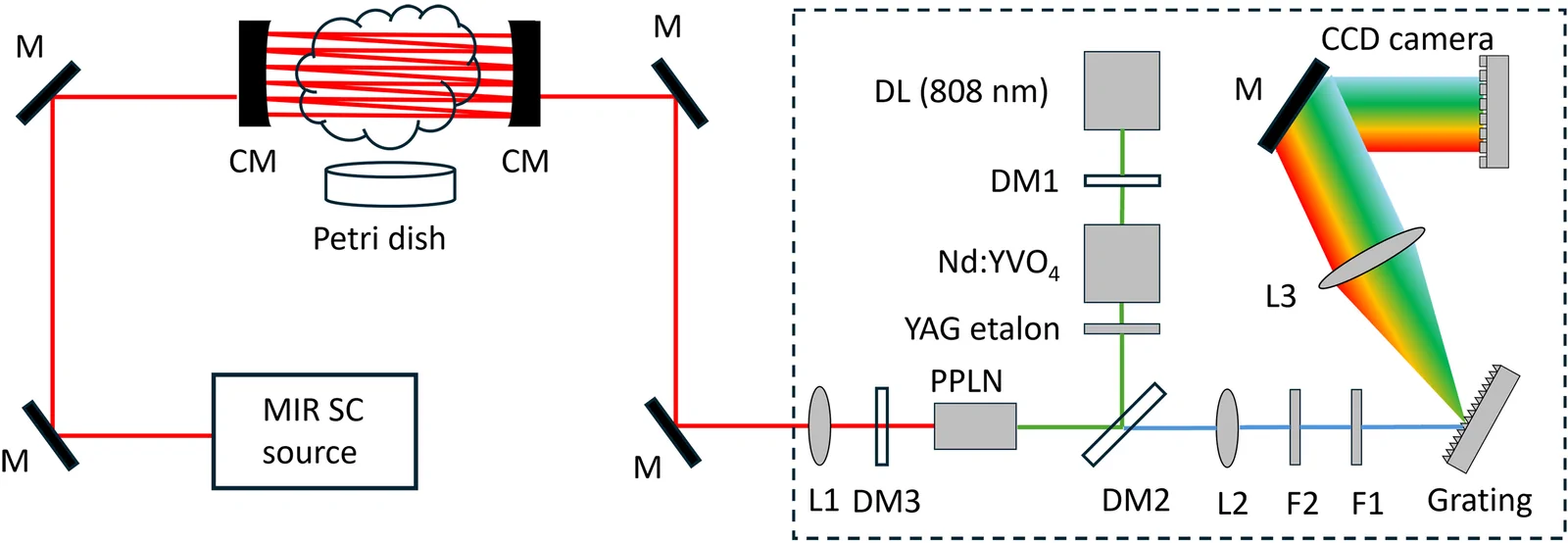
Schematic representation of the experiment system. M: mirror, CM: concave mirror, DL: diode laser, DM: dichroic mirror, Nd:YVO_4_: neodymium-doped yttrium vanadate gain media, PPLN: periodically poled lithium niobate, YAG etalon: temperature-stabilized etalon, L1: lens (*f* = 35 cm), L2: lens (*f* = 20 cm), L3: lens (*f* = 10 cm), F1: 750 nm long-pass filter, F2: 1000 nm short-pass filter.

The schematic design of the experiment system is shown in Figure 1. A broadband MIR SC source (SuperK MIR, NKT Photonics) was used as the light source, which delivered a total power of 320 mW within the spectral range of 1.8–4.1 μm and operated at a repetition rate of 2.5 MHz. The beam of the SC source was directed to an open Herriott-type multipass cell, which consisted of two concave spherical mirrors with an off-axis hole (CM508-200EH4-M02, Thorlabs), each with a focal length of 20 cm. The mirrors were placed parallel to each other at a distance of 39 cm. With 98 passes, the open multipass cell had an optical pathlength of 38.2 m in total. A 6 cm-wide petri dish was placed under the cell, from which the VOCs could evaporate freely and interact with the laser beam. The beam was directed to a commercial MIR-to-NIR upconversion spectrometer (S2055, NLIR). The basic principle of the spectrometer has been outlined in previous work.^20,21^ Inside the spectrometer, a neodymium-doped yttrium orthovanadate (Nd:YVO_4_) crystal, excited by a diode laser (808 nm), generated a 1064 nm continuous-wave pump laser beam. The input MIR beam was focused by a germanium lens (*f* = 35 mm, L1) on a periodically poled lithium niobate (PPLN) crystal, where it interacted with the 1064 nm laser beam and was upconverted through sum-frequency generation to NIR (750–1000 nm). The effective spectral coverage of the spectrometer was 3.1 to 4.2 μm, which was determined by the poling period of the PPLN crystal. A fixed NIR diffraction grating dispersed the upconverted beam into a silicon-based charge-coupled device (CCD) line camera (S11510-1006, Hamamatsu). The digital output of the line camera was transferred to a PC with a time resolution of up to 27 ms per spectrum. In this work, all of the recorded spectra were averaged up to 128 ms to optimize the signal-to-noise ratio. It should be noted that the spatial distribution of the NIR beam on the line camera was uneven because of the grating, which led to a non-uniform spectral resolution. For simplicity, we assumed a fixed spectral resolution of 6 cm^–1^.

Acetone (purity: 99.8%, VWR Chemicals BDH), ethanol (purity: 99.8%, VWR Chemicals BDH), and α-pinene (purity: 98%, Sigma-Aldrich) were selected to evaluate the performance of the system. The three compounds were monitored individually, and their evaporation dynamics were recorded at room temperature, as well as for a mixture of 0.5 ml of ethanol, 1 ml of acetone, and 2 ml of α-pinene. The evaporation of this mixture was also monitored at 40 °C. The petri dish was heated up by placing a high-power resistor (R = 68 Ω ± 5%, WH50, TT Electronics/WELWYN) with a 5 × 3 cm^2^ metal block under it. The resistor was connected to a power supply (PSD30/3B, BSI), and a thermocouple was connected to the metal block to monitor the temperature. All of the experiments followed the same procedure: no liquid sample was present during the initial 60 seconds to measure a background signal, after which the sample was injected into the petri dish. The petri dish was cleaned between different experiments to prevent contamination.

Obtaining a background spectrum without any absorption features is complicated by the open multipass cell design, as evacuation of the cell or purging with dry nitrogen (N_2_) gas in the open area is impossible. Thus, we took the first measured intensity spectrum without the presence of evaporating compounds as the background spectrum. All measured spectra were normalized to the background spectrum to generate absorbance spectra. In this way, the interfering absorption features from the ambient air (e.g., methane or water) were removed from the absorbance spectra. To monitor ambient CH_4_ and H_2_O, we generated a background spectrum by applying linear interpolation to the local maxima of the measured intensity spectrum, which were assumed to correspond to non-absorbing regions. The measured intensity spectrum was then normalized to the generated background and converted to an absorbance spectrum. The absorbance spectra were then fitted to reference spectra calculated based on the Pacific Northwest National Laboratory (PNNL) or HITRAN database^23,24^ parameters, convoluted with a Gaussian line shape function, to retrieve the concentration. For removing the baseline drifts, a polynomial (up to the fourth order) was fitted to the measurement together with the modelled spectra. As the VOCs can move freely within the open area, the distribution of VOCs is uneven, which makes it difficult to determine their concentrations. Therefore, the concentration of VOCs in this article is expressed as a product of volume fraction and interaction path length, indicated with a unit of parts per million per meter (ppm·m).

## Results

A series of experiments was conducted to evaluate the system's performance. First, the evaporation of each pure compound was monitored individually, and their concentration dynamics were recorded to demonstrate detection selectivity and unambiguous identification. Simultaneously, we show that, in addition to targeting broadly absorbing, high-purity VOC plumes, the system can also retrieve ambient concentrations of trace-level CH_4_ and H_2_O with narrow absorption features. Finally, a mixture of the three compounds was measured, and its evaporation behavior was investigated to assess the system’s capability for real-time multi-species detection and to demonstrate its applicability.

**Figure 2.**
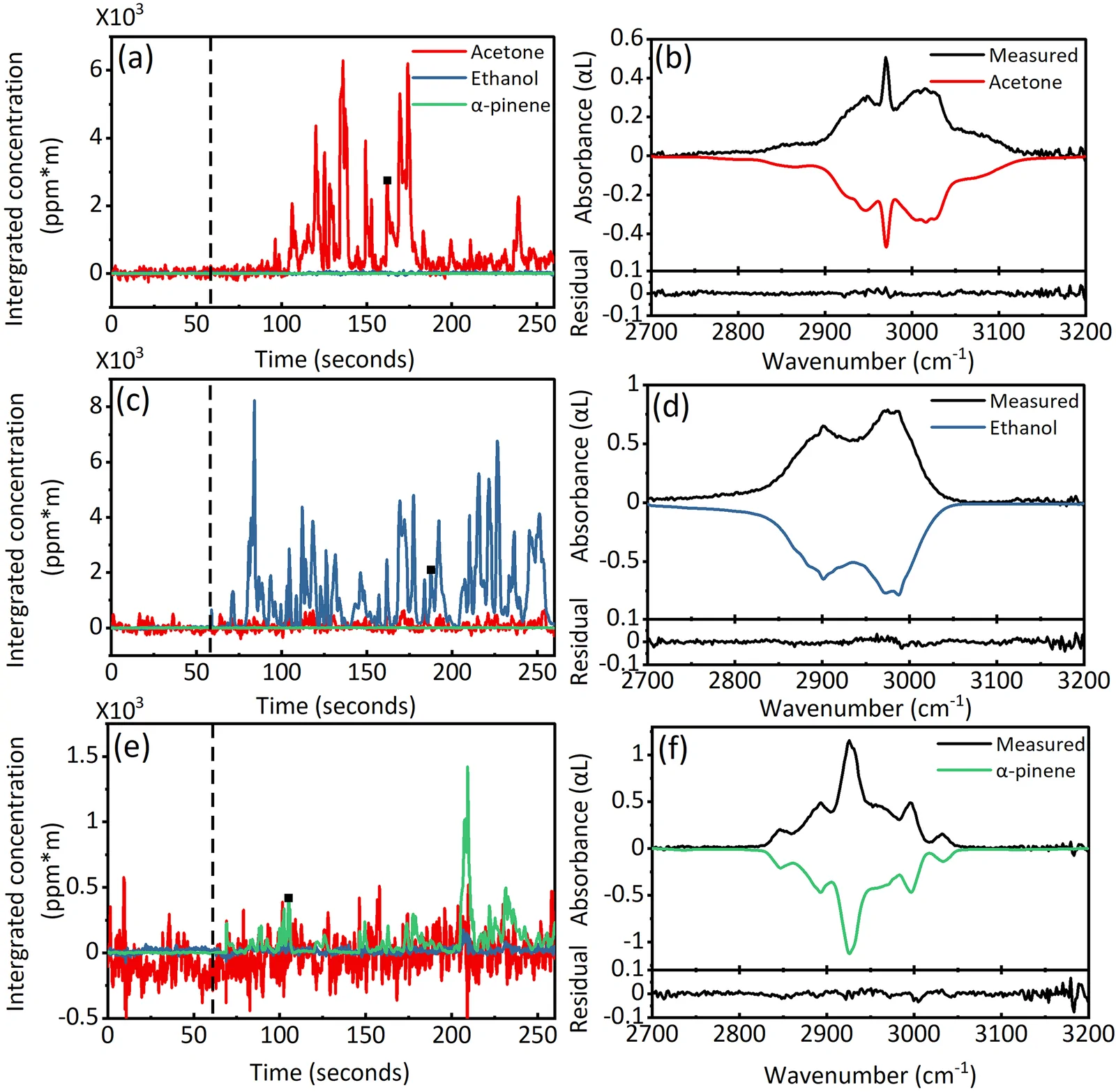
(a), (c), and (e) Retrieved concentration of acetone (red), ethanol (blue), and α-pinene (green) along with the measurement time for release of pure acetone, ethanol, and α-pinene, respectively. (b), (d), and (f) Measured absorbance spectrum (black) in 128 ms, with inverted fitted spectrum based on the PNNL database of acetone (red), ethanol (blue), and α-pinene (green), and the residual of the fit in the bottom panel.

To demonstrate that the system can distinguish different VOCs with overlapping absorption features in the C–H stretch functional group window (2850–3300 cm^–1^), we performed three separate measurements tracing the evaporation of each pure compound. It should be noted that three compounds were all included in the fitting model to evaluate the cross-interference effects during the measurements. Figures 2a, c, and e show the retrieved concentrations of three compounds (acetone in red, ethanol in blue, α-pinene in green) against the measurement time for the evaporation of pure acetone, ethanol, and α-pinene, respectively. During the first 60 seconds, no liquid compound was present on the petri dish. After ∼60 s (black dashed line), the compound was injected into the petri dish. The evaporation of this compound was recorded and displayed in Figures 2a, c, and e. The measured dynamics reflect the combined effects of evaporation and indoor turbulence. In the same panel, the retrieved concentrations of the other two compounds are also shown. The retrieved concentrations of the absent compounds are noise-limited and within the limit of detection, demonstrating the system’s cross-interference-free performance. The fitting procedure correctly identifies the typical absorption spectra of the three VOCs (indicated by black square dots in each retrieved concentration subplot) over the range of 2700–3200 cm^–1^ (Figures 2b, d, and f, with inverted PNNL-based reference spectra for clarity, respectively). The residuals, shown in the bottom of each panel exhibit no remaining absorption features, indicating the high quality of the fitting. These results further confirm the system’s ability to achieve unambiguous identification. The detection limits of the system, determined by its precision, were obtained from the noise-equivalent concentrations during the first 60 seconds of the experiment, before the respective compounds were introduced. For acetone, ethanol, and α-pinene, the detection limits were 30 ppm·m, 5 ppm·m, and 0.6 ppm·m in 1 second, respectively.

**Figure 3.**
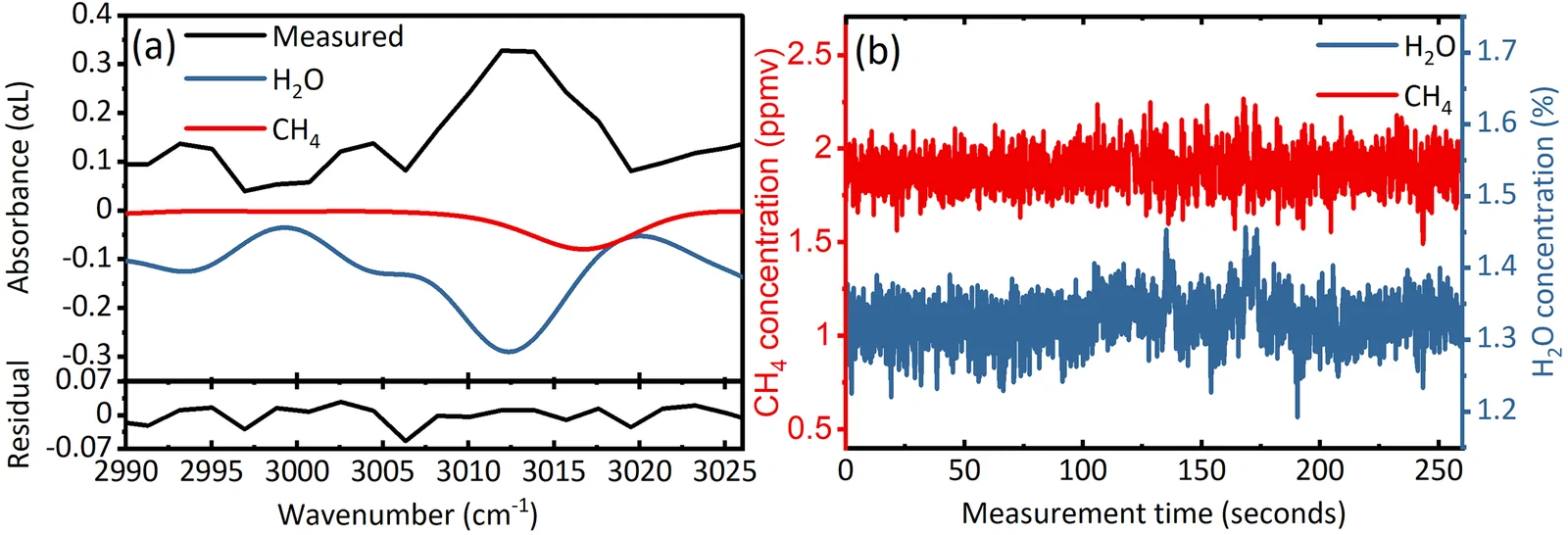
(a) Measured spectrum in 128 ms, with fitted spectrum based on HITRAN of H_2_O (blue) and CH_4_ (red) shown inverted and the fitting residual in the bottom panel. (b) Retrieved concentration of H_2_O and CH_4_.

We further investigated the CH_4_ and H_2_O concentrations during the measurement. A fraction of the spectrum, ranging from 2990 to 3026 cm^–1^ (2% of the CCD array), was selected for fitting, as this contains the CH_4_ Q-branch, which is its strongest peak. The retrieved results for CH_4_ and H_2_O are shown in Figure 3 for the data of the acetone evaporation (Figure 2a). In Figure 3a, the measured spectrum at a measurement time of 128 ms is shown (in black), along with inverted HITRAN-based simulated spectra of H_2_O (blue) and CH_4_ (red). Even with a coarse spectral resolution of 6 cm^–1^, the model still provides a good fit to the overlapping CH_4_ and H_2_O features, yielding a flat residual. The retrieved concentrations of both gases are displayed in Figure 3b. Average retrieved concentrations of 1.9 ± 0.08 ppmv for CH_4_ and 1.32 ± 0.03% for H_2_O (assuming a uniform distribution of these gases in the open cell) in 128 ms were obtained, where the uncertainty was based on the standard deviation of 100 consecutive 128-ms integrated measurements. The retrieved stable concentrations of both gases are consistent with the ambient laboratory conditions and underscore the robustness of the system and the multispecies detection capability: importantly, they remain unaffected by the concurrent plume of acetone passing through the system simultaneously (Figure 2 a).

**Figure 4.**
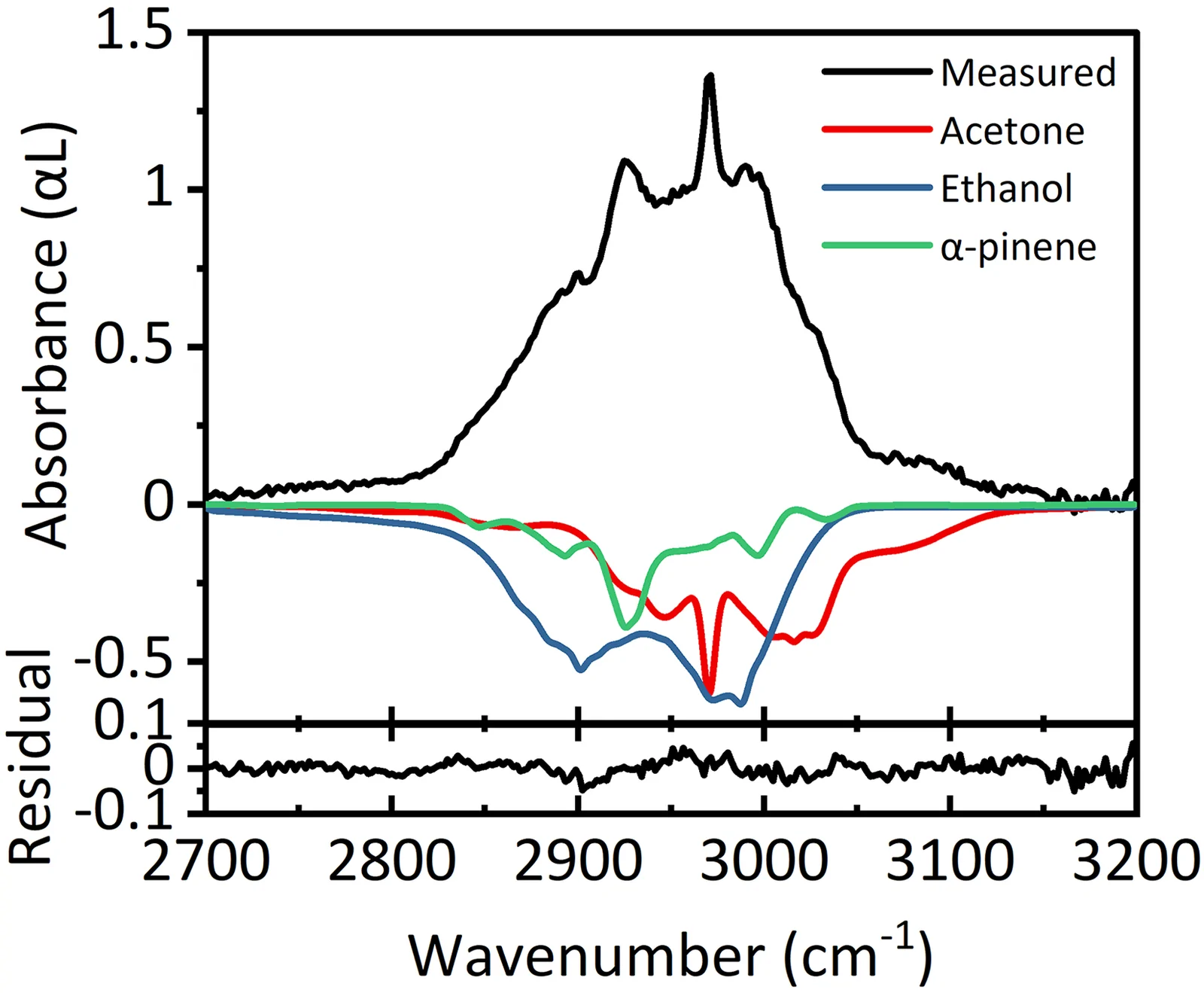
Absorbance spectrum (black) of a mixture of 0.5 ml ethanol, 1 ml acetone, and 2 ml α-pinene, measured in 128 ms, with the three inverted fitted spectra of acetone (red), ethanol (blue), and α-pinene (green) based on the PNNL database. The residual of the fit is shown in the bottom panel.

To further demonstrate the system's capability of multispecies detection, a mixture of 0.5 ml ethanol, 1 ml acetone, and 2 ml α-pinene was prepared and monitored during evaporation. A typical measured spectrum (black) of the evaporating mixture is plotted in Figure 4, along with the three inverted PNNL-based reference spectra (of ethanol, acetone, and α-pinene in blue, red, and green, respectively) for clarity. All three compounds show strong and overlapping absorption features with each other in the range between 2900–3000 cm^–1^. The broadband coverage of the source and spectrometer captures the characteristic spectral features of these compounds despite their overlap, enabling cross-interference-free quantification of these gas-phase VOCs, as shown in Figure 2. In a mixture, the fitting model successfully distinguishes the species and yields high-quality fits, as evidenced by the flat residual plot in the bottom panel.

**Figure 5.**
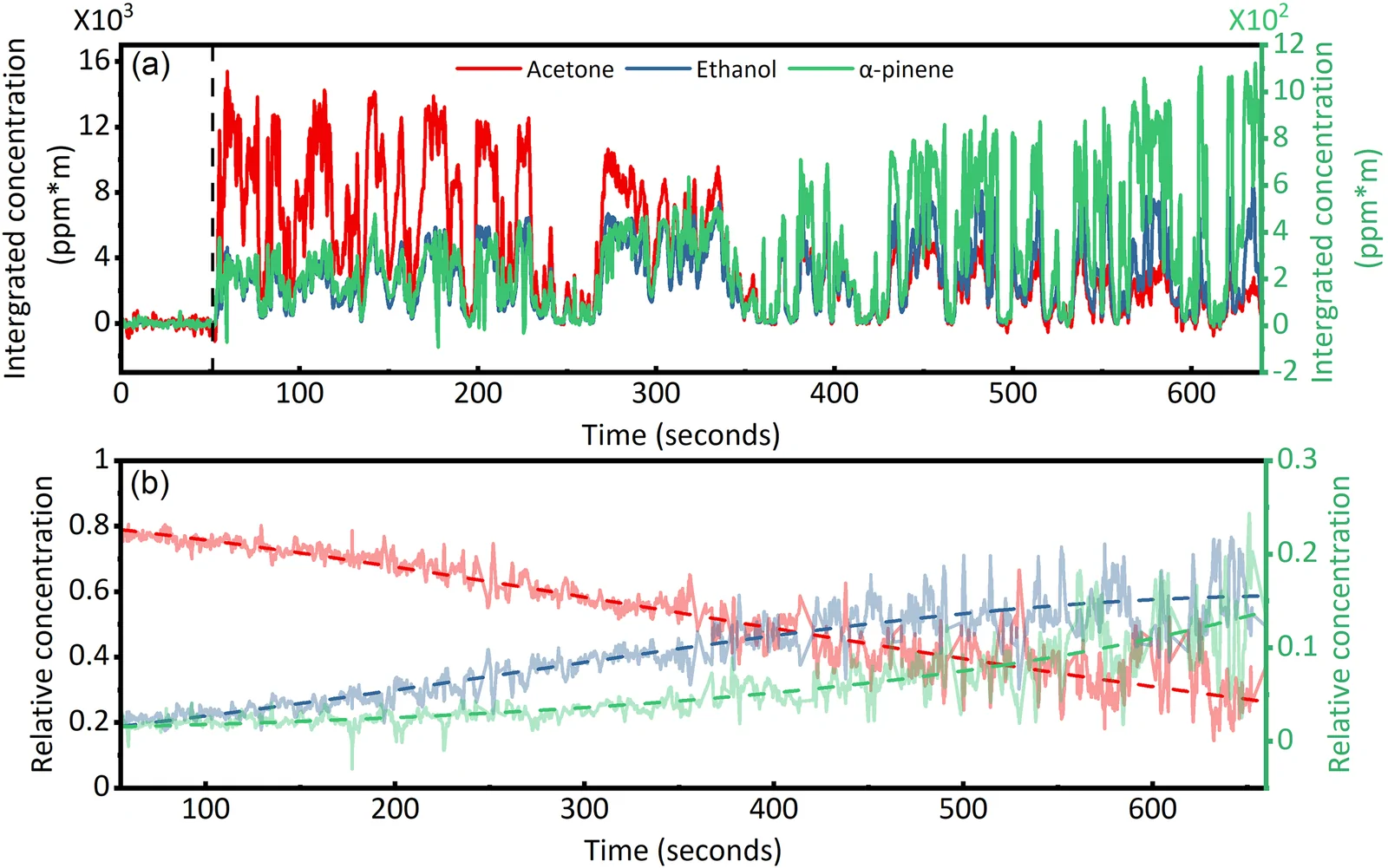
(a) Retrieved concentrations of acetone (red), ethanol (blue), and α-pinene (green). d(b) The relative ratio of retrieved concentrations of three compounds during the evaporation.

Measuring and quantifying the evaporation of a mixture of different compounds enable tracing of the evaporation dynamics over time. To show this, we monitored the mixture of 0.5 ml ethanol, 1 ml acetone, and 2 ml α-pinene as it was freely evaporating over time. The retrieved concentrations of the three compounds are shown in Figure 5a. As α-pinene is less volatile than the other two compounds, its retrieved concentrations are approximately one order of magnitude lower than the other two compounds, and the corresponding *y*-axis is thus shown on the right in green for clarity. In Figure 5a, the dynamic nature of the evaporation plume is apparent from the rough fluctuations in concentration over time: transient airflow fluctuations and turbulence above the sample cause the evaporating plume to pass the measurement instrument intermittently. However, calculating the relative ratio of the three compounds within the plume reveals a much more stable and consistent trend (Figure 5b). Over time, the ratio of the three compounds in the plume alters: initially, about 78% of the plume consists of acetone vapor, which decreases over time, while an increase of ethanol and α-pinene is observed. The dashed lines in Figure 5b indicate a guide to the eye for the relative increase or decrease over time per compound. The results are in agreement with the compounds’ vapor pressure, which is ∼533 mbar,^25^ ∼173 mbar,^26^ and ∼ 14 mbar^27^ for acetone, ethanol, and α-pinene, respectively, supporting the findings of the initial concentration ratios inside the plume, as well as the changing ratio over time, as acetone evaporates faster and thus is depleted towards the end of the measurement. These findings further consolidate the accurate multi-species detection and quantification of our measurement instrument for mixtures of VOCs with overlapping absorption features.

## Discussion

As shown in the previous results, the mid-IR SC source combined with the upconversion spectrometer and free-space multipass interaction path has the capability of fast, sensitive, and multispecies detection of evaporating VOCs. The detection of single-species, pure liquid compounds confirmed correct chemical identification while recording the evaporation dynamics at a time resolution of 128 ms, highlighting the system’s fast and cross-interference-free performance. The rapid VOC detection and identification make the system suitable for applications in occupational and indoor exposure. The simultaneous retrieval of CH_4_ and H_2_O concentrations in ambient air with high accuracy and high precision extends the system’s ability for multispecies detection even to compounds at environmental background concentrations. Such accuracy is remarkable, given the spectrometer’s coarse spectral resolution (6 cm^–1^) and the narrow spectral features of these compounds. Therefore, the reliable retrieval provides further confidence in the results obtained using this system. The evaporation of the mixture established that the three compounds were identified and monitored correctly, despite their strong spectral overlap. Their relative concentrations in the plume matched the expected ratios given by the compounds’ vapor pressures, underscoring the system’s accuracy for identification of the overlapping features.

Our system, based on mid-IR upconversion spectroscopy, invites comparison to the emerging field of mid-IR quantum, or “undetected-photon”, spectroscopy.^28,29^ This approach uses one nonlinear crystal for both the generation of mid-IR light and the upconversion to near-IR for detection, in contrast to our system, which consists of two separate nonlinear conversion steps (one in the mid-IR SC generation and one in the spectrometer). While quantum Fourier-transform infrared spectroscopy (QFT-IR) elegantly reduces the number of components compared to our solution, and thus provides a cost-effective alternative, the decoupling of the nonlinear steps in our approach significantly reduces the experimental complexity. However, current QFT-IR implementations require Fourier transform spectral reconstruction through mechanical optical path difference scanning, which constrains the temporal resolution to the second or minute timescale.^30–32^ For applications in occupational and indoor human VOC exposure monitoring, where concentration dynamics, i.e., leaks, can evolve on sub-second timescales, such temporal resolution is insufficient. The combination of a broadband MIR SC source with upconversion spectroscopy demonstrated in this work circumvents this limitation, achieving millisecond-scale spectral acquisition through parallel CCD-based detection without interferometric scanning. Moreover, our retrieved detection limits of 30 ppm·m, 5 ppm·m and 0.6 ppm·m in 1 second for acetone, ethanol, and α-pinene, respectively, are nearly one to two orders of magnitude better than a recent study by Neves et al., who used QFT-IR for the open-path detection of acetone and ethanol vapours.^30^ This demonstrates the much higher sensitivity of our approach, despite the similar power levels (∼few watts) of the pump diodes in the upconverter and QFT-IR.

Further advances of the system could primarily be achieved by broadening the spectral window. The current spectral window is 3.1 to 4.2 μm and strongly depends on the poling period and transmission window of the PPLN crystal. The spectral window could be broadened by using crystals from different materials or with different poling periods. Besides, the upconversion efficiency is restricted by the angle of the input MIR beam. Improved alignment of the system can increase the efficiency of upconversion and signal-to-noise ratio and potentially improve the time resolution (due to shorter required averaging times).

Most of the current commercial VOC sensors used in indoor exposure monitoring have a slow response time of seconds, tens of seconds, or even minutes.^33^ With a time resolution of 128 ms, our system can build a real-time alarm for indoor human VOC exposure accidents. Beyond alarm functions, the ability to identify and simultaneously retrieve multiple VOCs, together with background species (CH_4_ and H_2_O), provides the opportunity of multi-source attribution and compound-specific exposure tracking in complex indoor environments. Looking forward, indoor VOCs monitoring may no longer be limited to source identification and alarming but may evolve toward real-time spatial mapping of indoor VOC plumes. Ultimately, the development of these capabilities can lead toward a future intelligent, self-controlled indoor monitoring network that combines fast, reliable sensing, autonomous interference suppression, and data-driven modeling to support healthier indoor environments and more responsive exposure-mitigation strategies.

## Conclusion

Combining a broadband MIR SC source, a free-space multipass interaction path, and a grating-based upconversion spectrometer enables fast, sensitive, and multi-species detection of evaporating VOCs. Upconversion spectroscopy overcomes the shortcomings of conventional grating-based spectroscopy in the MIR range by achieving a time resolution of 128 ms, which can be used to trace the dynamics of each compound during evaporation. The free-space multipass interaction path or “open multipass cell” alignment not only prevents effects of contamination and adsorption but also enables direct observation of the turbulent plume of evaporating compounds through the beam path. The system provides cross-interference-free measurements of both complex mixtures with overlapping absorption features and ambient compounds at high sensitivity, achieving the detection limits of 30 ppm·m, 5 ppm·m, and 0.6 ppm·m in 1 second for acetone, ethanol, and α-pinene, and 0.03 ppm and 0.02% in 1 second for CH_4_ and H_2_O, respectively. These results pave the way for applications in occupational and indoor exposure, such as real-time alarm systems for VOC exposure.
